# Dynamic alteration and prognostic significance of tumor‐associated CD68^+^ and CD68^+^PD‐L1^−^ macrophages in muscle‐invasive bladder cancer treated with neoadjuvant chemotherapy

**DOI:** 10.1002/cam4.5191

**Published:** 2022-08-31

**Authors:** Jie Wu, Rui‐Yang Xie, Li‐Hui Wei, Chuan‐Zhen Cao, Bing‐Qing Shang, You‐Yan Guan, Hong‐Zhe Shi, Wang Qu, Yun Li, Jing Liang, Shan Zheng, Ai‐Ping Zhou, Xiao‐Feng Zhou, Jian‐Zhong Shou, Xin‐Gang Bi

**Affiliations:** ^1^ Department of Urology National Cancer Center/National Clinical Research Center for Cancer/Cancer Hospital, Chinese Academy of Medical Sciences and Peking Union Medical College Beijing China; ^2^ Genecast Biotechnology Co., Ltd Wuxi Jiangsu China; ^3^ Department of Urology China‐Japan Friendship Hospital Beijing China; ^4^ Department of Medical Oncology National Cancer Center/National Clinical Research Center for Cancer/Cancer Hospital, Chinese Academy of Medical Sciences and Peking Union Medical College Beijing China; ^5^ Department of Pathology National Cancer Center/National Clinical Research Center for Cancer/Cancer Hospital, Chinese Academy of Medical Sciences and Peking Union Medical College Beijing China

**Keywords:** multiplex immunofluorescence staining, muscle‐invasive bladder cancer, neoadjuvant chemotherapy, prognosis, tumor‐associated macrophages

## Abstract

**Background:**

The current study aimed to investigate the dynamic alteration and prognostic significance of tumor‐infiltrating lymphocytes (TILs), tumor‐associated macrophages (TAMs), and PD‐L1 status of immune cells in muscle‐invasive bladder cancer (MIBC) treated with neoadjuvant chemotherapy (NAC).

**Methods:**

Multiplex immunofluorescence staining was performed to examine CD68^+^ TAM, CD4^+^ T cell, CD8^+^ T cell, FOXP3^+^ Treg cell, and PD‐L1 expression in paired MIBC tissues (*n* = 54) before and after NAC. Patients were then divided into definite responders (DR), (≤pT1) and incomplete responders (IR).

**Results:**

There was no significant difference between DR and IR cohorts for the immune cell infiltration levels at the baseline status. Tobacco history was identified to be associated with worse NAC efficacy. CD68^+^ (stroma area: *p* = 0.025; tumor area: *p* = 0.028; total area: *p* = 0.013) and CD68^+^PD‐L1^−^ (stroma area: *p* = 0.035; tumor area: *p* = 0.013 total area: *p* = 0.014) TAMs infiltration levels decreased significantly after NAC, while there was no significant difference of CD68^+^PD‐L1^+^ and TILs. The infiltration of CD68^+^ (*p* = 0.033), CD68^+^PD‐L1^−^ (*p* = 0.033), and CD68^+^PD‐L1^+^ (*p* < 0.001) TAMs in stroma area were significantly associated with poorer disease‐free survival rate (DFS) of MIBC patients.

**Conclusion:**

CD68^+^ and CD68^+^PD‐L1^−^ TAMs infiltration levels decreased significantly after NAC and pre‐treatment TAM infiltration levels were independent prognostic factors for MIBC patients. While there was no sufficient evidence demonstrating that pre‐treatment TILs or TAMs could predict response to NAC in MIBC patients.

## Graphical abstract

In this study, we performed multiplex immunofluorescence to test CD68^+^TAMs, FOXP3^+^ Treg cell, CD4^+^ T cell, CD8^+^ T cell, and PD‐L1 expression in MIBC before and after NAC. The results revealed that CD68^+^ and CD68^+^PD‐L1^−^ TAM infiltration levels decreased significantly after NAC and pre‐treatment TAM infiltration levels were independent prognostic factors for MIBC patients. However, there was no sufficient evidence demonstrating that pre‐treatment TILs or TAMs could predict response to NAC in MIBC patients.

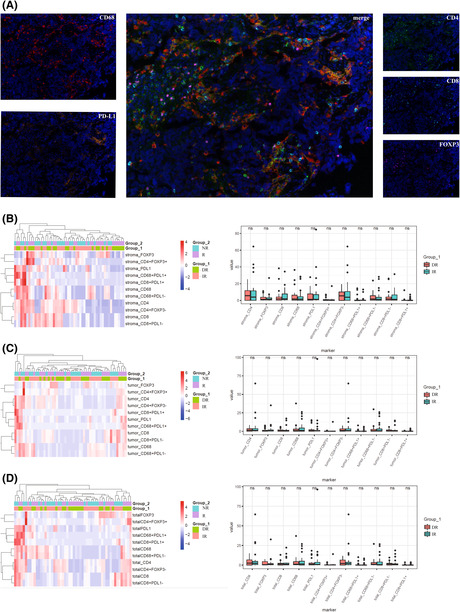

## 1 BACKGROUND

Bladder cancer is the twelfth most commonly diagnosed malignancy worldwide and muscle‐invasive bladder cancer (MIBC) represents about 20% with early metastasis and a high mortality.^1^, ^2^, ^3^ Cisplatin‐based neoadjuvant chemotherapy (NAC) combined with radical cystectomy (RC) or selective bladder preservation therapy are preferred regimens for MIBC. However, NAC was underused predominantly because the clinical benefit was restricted to a subset of complete responders accounting for 30%–40%, and there were no validated molecular markers or clinical characteristics to adequately screen the patients who could benefit from NAC.^4^, ^5^ How to predict NAC responsiveness remains a major challenge at present. As next‐generation sequencing technologies have improved over the last decades, some molecular markers predicting NAC responsiveness were studied, such as ERCC2, ATM, RB1, FANCC, and ERBB2.^6^, ^7^, ^8^, ^9^ However, given these studies' retrospective nature or lacking randomization between NAC and cystectomy alone, the clinical validity and utility of these markers in informing NAC decisions remain unclear. Except for the discovery of specific molecular markers correlated with NAC responsiveness, several molecular classification systems of urothelial cancer have been identified and associated with NAC response, including Lund University system, MD Anderson Cancer Center system, and the cancer genome atlas (TCGA) system.^10^, ^11^, ^12^ It has been previous reported that patients with basal tumors had better response to NAC, while patients with immune‐infiltrated luminal subtype had limited benefit from NAC, indicating that immune infiltrating may also be associated with the efficacy of NAC.^13^

The roles of various immune cells in the tumor microenvironment (TME) are complicated and essential in cancer therapy. The majority of immune cells in bladder cancer (BC) are tumor associate macrophages (TAMs), tumor‐infiltrating lymphocytes (TILs), and myeloid‐derived suppressor cells.^14^ TILs, including CD4^+^ T cell, CD8^+^ T cell, and Treg cell, cooperate with tumor cells through the release of chemokines and cytokines acting as important tumorigenic and prognostic factors.^15^ TAMs play an essential role in solid tumor development, and have been demonstrated as a significant factor in tumor growth and progression in several cancers, including MIBC.^16^, ^17^ However, the interrelation between TAMs/TILs and prognosis in MIBC treated with NAC has not been fully elucidated, and the TAMs data were all baseline status without comparing between pre‐NAC and post‐NAC. Moreover, the immune checkpoint inhibitors (ICIs) have deeply altered the therapeutic paradigm of BC and PD‐L1 status on tumor cells has been identified as a predictor for ICI responsiveness.^18^ Recently, PD‐L1 expression in tumor‐infiltrating immune cells has been proven as a prognostic factor in urothelial carcinoma.^19^, ^20^ While the predictive value of tumor‐infiltrating immune cells PD‐L1 status failed to be systematically explored in MIBC patients treated with NAC.

In this study, simultaneous testing of CD68^+^TAMs, FOXP3^+^ Treg cell, CD4^+^ T cell, CD8^+^ T cell, and PD‐L1 expression in MIBC before and after NAC were conducted by multiplex immunofluorescence technique.

## 2 METHODS

### 2.1 Patients' selection and treatment strategy

The current study initially enrolled 101 patients with MIBC (T2‐4aN0M0), who had received NAC at our center from January 2012 to December 2019. Three to four cycles of Gemcitabine/Cisplatin regimen NAC. The efficacy of NAC was defined below. Patients with significant down‐staging (≤T1) were categorized as definite responders (DR). Incomplete responders (IR) were defined as patients whose primary tumors were not significantly down‐staged. Patients were defined as having progressive disease (PD) when imaging examinations revealed that a new lesion had appeared, or the maximum diameter of the primary tumor had increased by more than 20%. Whereafter, DR and IR received definite treatment within 2 months. Imaging examinations, blood sampling, and physical examination were performed every 3 months for 2 years after definite treatment, then every 6 months in the 3 year and annually afterwards. Patients who were enrolled in the final cohort need to accord with the following key inclusion criteria: (1) paired tumor tissues before and after NAC were available; (2) two pathologists confirmed the pre‐treatment pathologies were all urothelial carcinoma and post‐treatment T stage; (3) no other malignant tumors or chemotherapy history; (4) complete follow‐up information (Figure S1). This study was approved by the Ethics Committee of the National Cancer Center/Cancer Hospital, Chinese Academy of Medical Sciences (approval number: NCC2015 YL‐05). Written informed consent was included in each medical record.

### 2.2 Multiplex immunofluorescence staining

We used multiplex immunofluorescence staining to examine the tumor immune microenvironment of samples from MIBC patients. The primary antibodies used in this study were as follow: CD4 antibody (clone EP204, ZA0519, Zsbio), CD8 antibody (clone SP16, ZA0508, Zsbio), CD68 antibody (clone KP1, ZM0060, Zsbio), FOXP3 antibody (clone 236A/E7, ab20034, abcam), and PD‐L1 antibody (clone E1L3N, CST13684, CST). All the antibodies were diluted in 1:100 except for CD68 antibody, the dilution of which was 1:500. Tumor tissue of fixed paraffin embedded was collected from bladder surgeries before and after NAC, and the thickness of each slide was 4‐μm. After deparaffinized in xylene, rehydrated, and washed in tap water, the slides were boiled in Tris‐EDTA buffer (pH 9; 643,901; Klinipath) for epitope retrieval/microwave treatment (MWT). In order to block endogenous peroxidase, Antibody (Ab) Diluent/Block (72,424,205, PerkinElmer) was used then.

For the incubation of the primary antibody, CD4, CD8, CD68, FOXP3, and PD‐L1 were incubated for 1 h at 37°C. The secondary antibody incubation was performed at 37°C for 10 min with Opal Ploymer horseradish peroxidase (HRP) Ms + Rb (2,414,515; PerkinElmer). After the incubations were finished, tyramide signal amplification (TSA) visualization with the Opal seven‐color immunohistochemistry Kit (NEL797B001KT; PerkinElmer) and TSA Coumarin system (NEL703001KT; PerkinElmer) were performed. The next antibody was labeled by repeating the steps above. After all the antibodies were labeled, MWT with Tris‐EDTA buffer (pH9) was proceeded to remove the antibody TSA complex. TSA single stain slides were finished with MWT and counterstained with 4,6‐diamidino‐2‐phenylindole (DAPI) for 5 min and were enclosed in Antifade Mounting Medium (I0052; NobleRyder).

After the above steps were finished, PerkinElmer Vectra (Vectra V.3.0.5, PerkinElmer) was used to scan the slides and inForm Advanced Image Analysis software (inForm V.2.3.0, PerkinElmer) was used to analyze each marker in stained cells. We used mean fluorescence intensity (MFI) to quantify the marker expression in each stained cell membrane.^21^ As previously described, positive cells were defined as cells with true immunofluorescence signal detected (>median MFI of all stained cells in a slide), and the right expression pattern (FOXP3^+^ cells at cell nucleus, CD4^+^ and CD8^+^ cells at cell membrane, and PD‐L1^+^ cells at cell membrane and cytoplasm) was also necessary at the same time. And 5–15 representative original multispectral images were selected to train the inform software (tissue segmentation, cell segmentation, phenotyping tool, and positivity score). We saved all the settings within an algorithm so that the following analysis of multiple original multispectral images from the same slide could proceed smoothly. Two experienced pathologists calculated the number, percentage, and density of positive cells under the ×200 magnification of each slide with more than 10 selected fields. The further analysis we used the percentage of positive cells in all nucleated cells of the tumor nests, tumor stroma and total region from the selected fields. In all, the infiltration levels of CD4^+^, CD8^+^, CD68^+^, FOXP3^+^, PD‐L1^+^, CD4^+^FOXP3^+^, CD4^+^FOXP3^−^, CD8^+^PD‐L1^+^, CD8^+^PD‐L1^−^, CD68^+^PD‐L1^+^, and CD68^+^PD‐L1^−^ were discussed in this research.

### 2.3 Bioinformatics analysis

The Tumor IMmune Estimation Resource (TIMER) algorithm database (https://cistrome.shinyapps.io/timer/) was utilized to investigate the prognostic values of CD4^+^ T cell, CD8^+^ T cell, and macrophage.

### 2.4 Statistical analysis

R software version 4.1.0 (http: //www.R‐project.org) and its corresponding packages were used to perform the statistical analysis. The disease‐free survival rate (DFS) was evaluated by using the Kaplan–Meier curve, and the log‐rank test was utilized for comparing different groups. The last follow‐up time was March 1, 2021. Logistic analysis was conducted to identify variables correlated with NAC efficiency and Cox proportional hazard regression analysis was utilized to identify the significant determinant factors for DFS. Comparisons between definite responder and incomplete responder group regarding immune cell infiltration levels were conducted by using paired *t* test, comparisons between pre‐ and post‐NAC treatment were conducted by using the Wilcox test, and statistical significance was defined as *p* < 0.05.

## 3 RESULTS

### 3.1 Immune profiling between DR and IR at baseline

**FIGURE 1:**
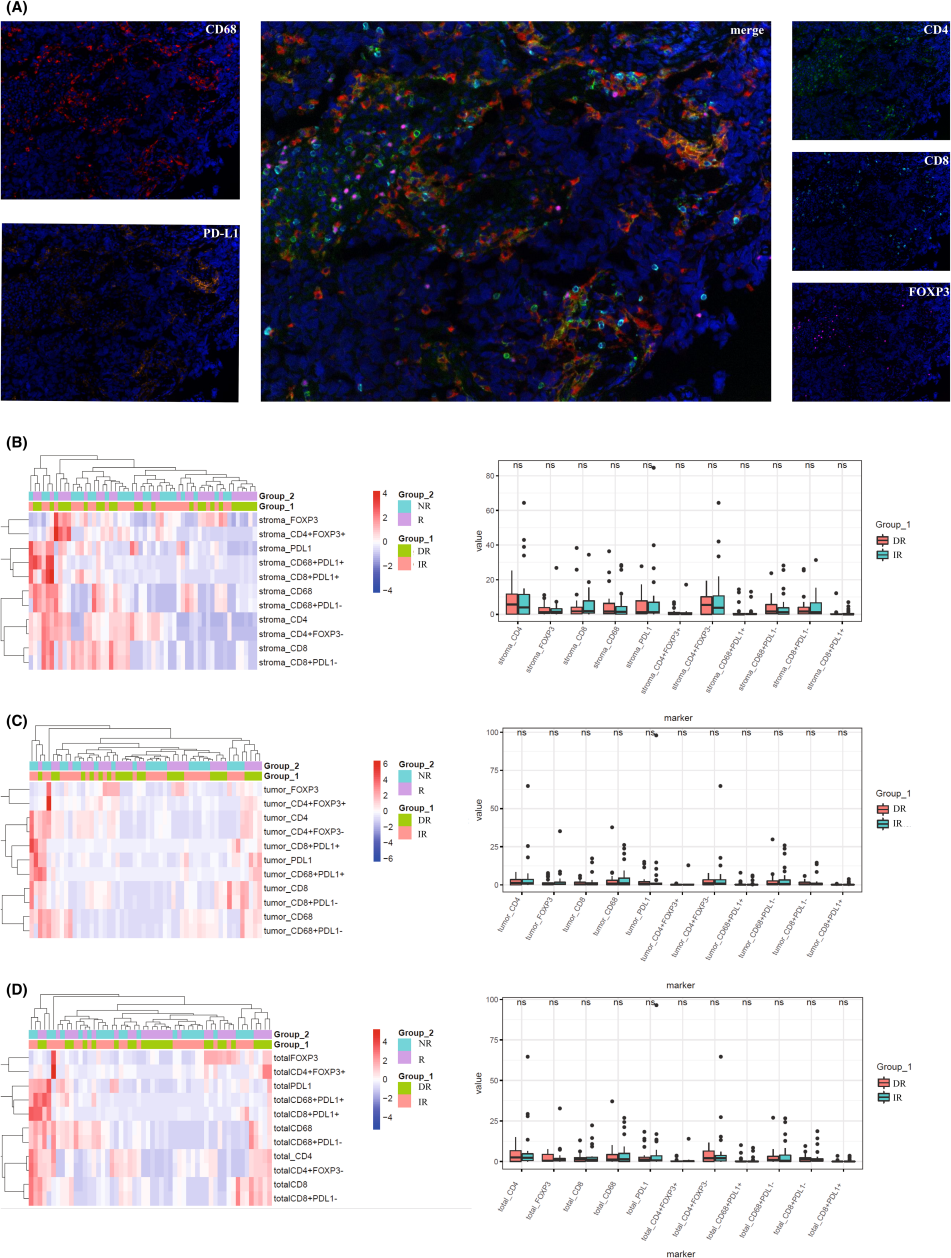
(A) Representative multiplex immunofluorescences: related markers including CD68^+^, CD4^+^, CD8^+^, FOXP3^+^, and PD‐L1^+^. Heatmaps and boxplots of percentage of positive cells of markers between group 1* and group 2: (B) stroma, (C) tumor, (D) total. *NAC efficacy evaluation based on the CT/MR according to the RECIST criterion: Non‐responder (NR): stable disease or progressive disease. Responder (R): complete responder or partial responder.

A total of 54 eligible patients were identified and all the patients were grouped according to the efficacy of NAC: DR (*n* = 25) and IR (*n* = 29). The cohort distribution and clinical data were shown in Table 1. We firstly tested immunologic markers of CD4^+^, CD8^+^, CD68^+^, FOXP3^+^, PD‐L1^+^, CD4^+^FOXP3^+^, CD4^+^FOXP3^−^, CD68^+^PD‐L1^+^, CD68^+^PD‐L1^−^, CD8^+^PD‐L1^+^, and CD8^+^PDL1^−^ in the tumor core area, tumor‐associated stroma area, and total area before NAC. However, there is no significant difference between DR and IR for the immune cell infiltration levels at the baseline status (Figure 1). Notably, based on logistic regression analysis, tobacco history was associated with poorer NAC efficacy (HR: 3.405, *p* = 0.038), and the predictive value to NAC of pretreatment neutrophil‐to‐lymphocyte ratio (NLR) was tended toward significance (HR: 3.824, *p* = 0.097) (Table S1).

**TABLE 1:** Demographic and clinical characteristics of MIBC patients who received NAC

Variable	All patients (*N* = 54)
Median age, years (range)	63 (35.0–73.0)
Gender, *n* (%)	
Male	47 (87.0)
Female	7 (13.0)
Median body mass index (BMI), kg/m^2^ (range)	24.2 (18.4–31.4)
Tobacco history, *n* (%)	
No	20 (37.0)
Yes	34 (63.0)
Pre‐treatment T stage, *n* (%)	
T2	13 (24.1)
T3	36 (66.7)
T4a	5 (9.2)
Lesion, *n* (%)	
Single	33 (61.1)
Multiple	21 (38.9)
Median value of tumor size, cm (range)	3.0 (2.0–6.0)
NAC cycles, *n* (%)	
3	39 (72.2%)
4	15 (27.8%)
NAC efficacy, *n* (%)	
Definite responder (≤T1)	25 (46.3%)
Incomplete responder	29 (53.7%)
Pathological T staging, *n* (%)	
pT1	25 (46.3%)
pT2	13 (24.1%)
pT3	13 (24.1%)
pT4	3 (5.6%)
Median follow‐up time, months	45.2
Tumor relapse after surgery, *n* (%)	
No	29 (53.7)
Yes	25 (46.3)
Vital status, *n* (%)	
Alive	37 (68.5)
Dead	17 (31.5)

Abbreviations: MIBC, muscle‐invasive bladder cancer; NAC, neoadjuvant chemotherapy.

### 3.2 Dynamic alterations of the immune cell infiltration levels pre‐ and post‐NAC treatment

**FIGURE 2:**
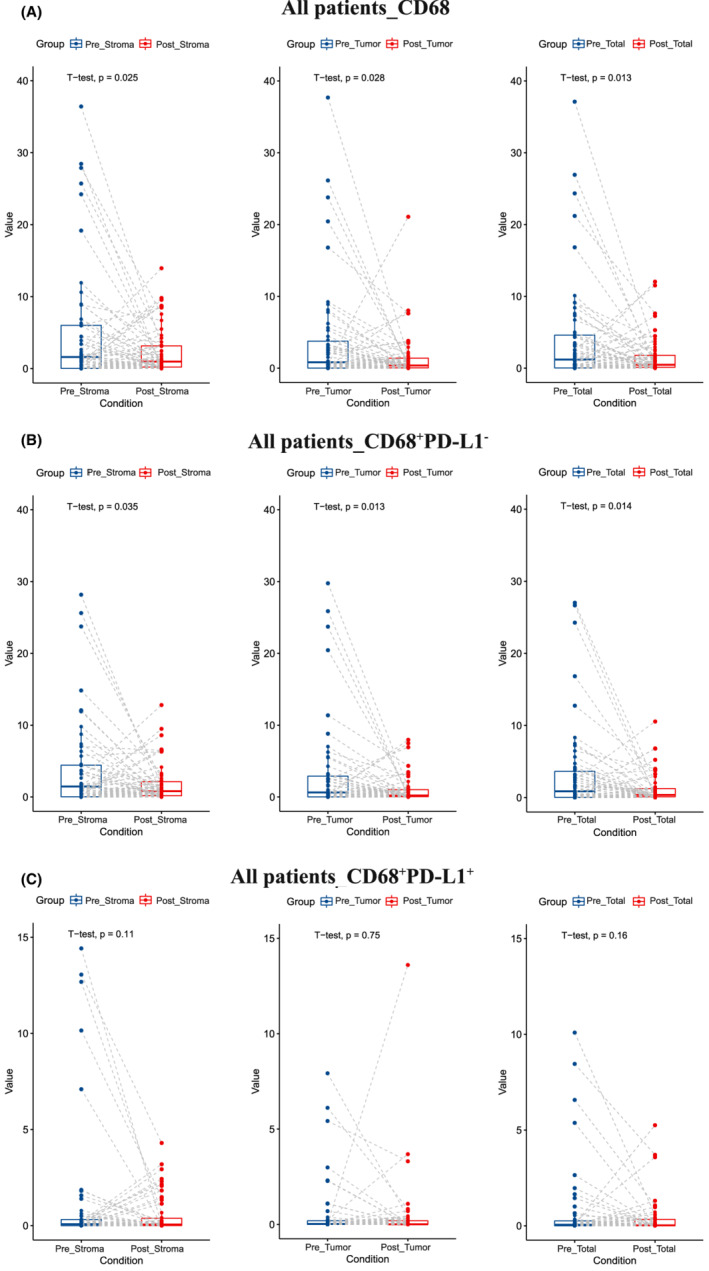
Paired analysis of before and after neoadjuvant chemotherapy in (A) CD68^+^, (B) CD68^+^PD‐L1^−^, and (C) CD68^+^PD‐L1^+^.

**FIGURE 3:**
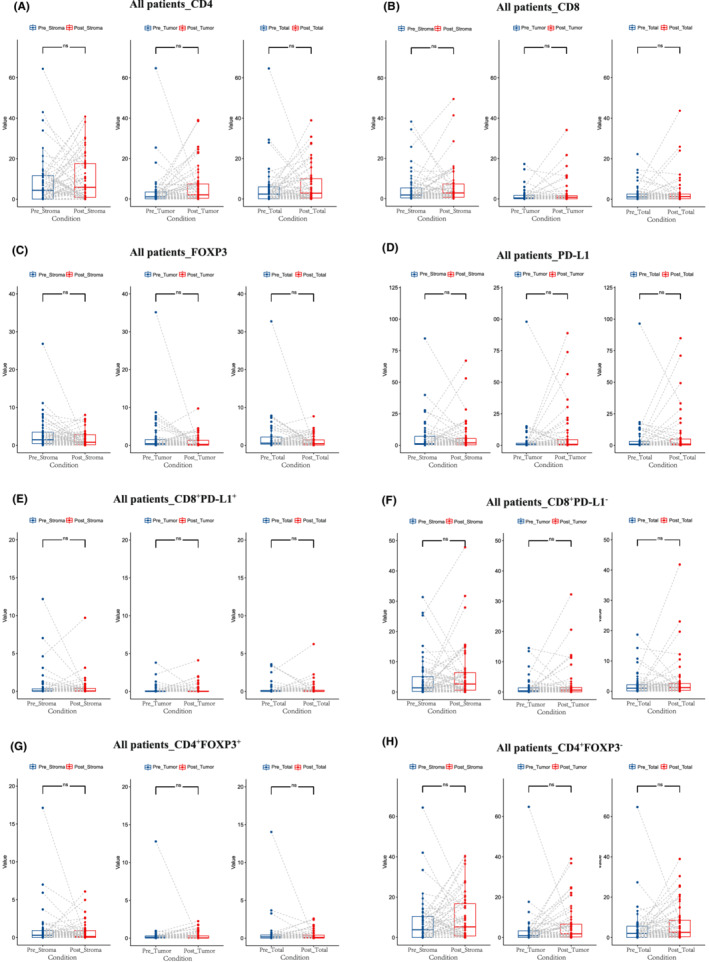
Paired analysis of before and after neoadjuvant chemotherapy in (A) CD4^+^, (B) CD8^+^, (C) FOXP3^+^, (D) PD‐L1^+^, (E) CD8^+^PD‐L1^+^, (F) CD8^+^PD‐L1^−^, (G) CD4^+^FOXP3^+^, and (H) CD4^+^FOXP3^−^.

We next investigated the alterations of the immune cell infiltration levels pre ‐ and post‐NAC treatment. Notably, CD68^+^ TAMs infiltration level decreased significantly after NAC (stroma area: *p* = 0.025; tumor area: *p* = 0.028; total area: *p* = 0.013) (Figure 2A). Among them, CD68^+^PD‐L1^−^ TAMs decreased significantly (stroma area: *p* = 0.035; tumor area: *p* = 0.013 total area: *p* = 0.014) (Figure 2B), while there was no significant difference of CD68^+^PD‐L1^+^ TAMs (stroma area: *p* = 0.11; tumor area: *p* = 0.75; total area: *p* = 0.16) (Figure 2C). The infiltration levels of CD4^+^ T cell, CD8^+^ T cell, or FOXP3^+^ Treg cell did not change significantly after NAC (Figure 3).

### 3.3 Prognosis values of tumor‐associated macrophages infiltration

**FIGURE 4:**
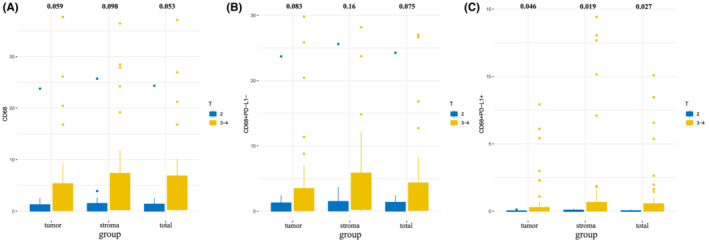
Boxplots of percentage of positive cells of (A) CD68^+^, (B) CD68^+^PD‐L1^−^, and (C) CD68^+^PD‐L1^+^ between groups of T2 and T3‐4.

**FIGURE 5:**
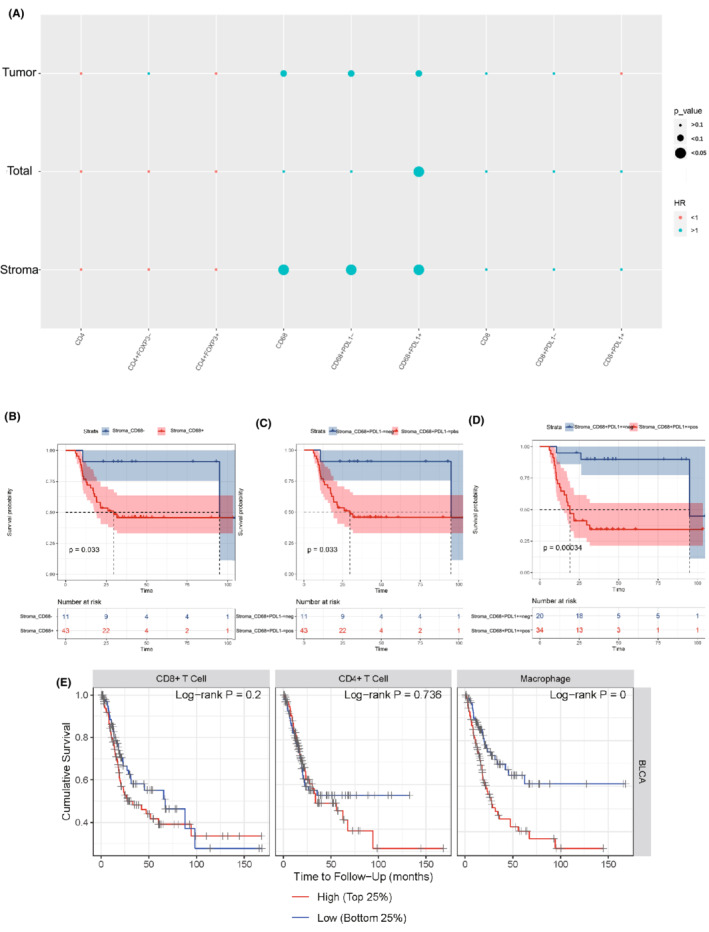
(A) Bubble plot illustrating the distribution of the HR on disease‐free survival of all the markers before neoadjuvant chemotherapy. Kaplan–Meier (K‐M) curves and risk tables of disease‐free survival based on the expression of (B) CD68^+^, (C) CD68^+^PD‐L1^−^, and (D) CD68^+^PD‐L1^+^. (E) K‐M curves of overall survival according to the infiltration level of immune cells based on TIMER database.

We then found that the patients of T3–T4 showed higher CD68^+^, CD68^+^PD‐L1^−^, and CD68^+^PD‐L1^+^ TAMs infiltration than those of T2 (Figure 4), indicating the correlation between TAMs and tumor invasion. We further investigated the correlations between the immune cell infiltration levels and survival outcome in MIBC patients. According to Cox regression analysis, the infiltration of CD68^+^, CD68^+^PD‐L1^−^, and CD68^+^PD‐L1^+^ TAMs in stroma area were significantly associated with poorer DFS (Figure 5A). The Kaplan–Meier curve and Log rank test analysis also revealed the prognosis values of stroma CD68^+^ (*p* = 0.033), CD68^+^PD‐L1^−^ (*p* = 0.033), and CD68^+^PD‐L1^+^ (*p* < 0.001) TAMs infiltration (Figure 5B–D). The results based on the TIMER database further demonstrated that TAM infiltration was an independent risk factor of poor outcomes for MIBC patients (Figure 5E). Notably, tumor immune microenvironment changed after NAC treatment and did not have prognosis value for MIBC (Figure S2).

## 4 DISCUSSION

MIBC is an aggressive disease requiring active management and MIBC patients have a high propensity to experience lymph node involvement and distant metastasis. NAC followed by RC is the standard treatment strategy of MIBC when surgery can be tolerated, supported by the updated European Association of Urology guidelines and the National Comprehensive Cancer Network (NCCN) guidelines as a Category 1 recommendation.^4^ Available evidence suggests that TME might play a crucial role in tumor progression and be associated with efficacy of NAC. Efstathioua et al. performed transcriptome‐wide gene profiling to investigate the role of immune signatures in MIBC and found that higher immune infiltration level is correlated with improved disease‐free survival in bladder‐sparing trimodality therapy (TMT) cohort, while higher stromal infiltration level is correlated with poorer disease‐free survival in NAC cohort.^22^

In the current study, we found that none of the immune cell markers (PD‐L1^+^, FOXP3^+^, CD68^+^, CD4^+^, CD8^+^) is reliable predictive factor to identify MIBC patients who are most likely to benefit from NAC. While it was previously reported by Ikarashi et al. that tumor‐infiltrating CD8^+^ T cells and CD204^+^ cells are correlated with the poor response of NAC in MIBC patients.^23^ The predictive value of immunologic markers to neoadjuvant chemotherapy efficacy in MIBC remains controversial. Our results further demonstrated that the infiltration of CD68^+^, CD68^+^PD‐L1^−^, and CD68^+^PD‐L1^+^ TAMs were significantly associated with poorer DFS of MIBC patients, while other TILs including infiltrating CD4^+^ and CD8^+^ T cells were not prognostic factors, which were also validated by TIMER database. Moreover, we observed that TAMs were positively correlated with high pathologic T stage. It is well‐known that the increased level of infiltrating CD8^+^ T cells is correlated with better prognosis in a number of human cancers, including breast cancer, colorectal cancer, and melanoma.^24^ However, the prognostic role of CD8^+^ T cells in bladder cancer is divergent according to our results, and individual studies exhibited contradictory results. Ikarashi et al. found that MIBC patients with high infiltrating CD8^+^ T cells level had worse prognosis in NAC cohort.^23^ The tumor‐infiltrating CD8^+^ T cell states exhibit a vast heterogeneity between patients, including cytotoxic CD8^+^ T cells and exhausted CD8^+^ T cells, and the further investigation of this heterogeneity is important to identify the association between individual T cell states and NAC response in MIBC patients.^24^, ^25^ In the microenvironment of solid tumors, TAMs are one of the most abundant infiltrating immune cells. Existing evidence reveals that the infiltration of TAMs is associated with unfavorable clinical outcomes in MIBC, consistent with our results.^16^, ^17^ Notably, TAMs can be divided into M1‐like (pro‐inflammatory) and M2‐like (anti‐inflammatory) TAMs based on their distinct phenotypes. During tumor progression, M1‐like TAMs are gradually derived toward a pro‐tumor M2‐like phenotype, and stimulate progression and invasion of tumor.^26^

Notably, tobacco history and NLR were identified to be associated with worse NAC efficacy in the current study. However, the values of smoking and NLR as predictive markers in MIBC patients receiving NAC remain controversial. Smoking has been demonstrated as the primary etiologic risk factor for bladder cancer and to participate in chemoresistance, while Kim et al. retrospectively analyzed 139 patients and found that there was no significant association between tobacco history and response to NAC.^27^ NLR is a well‐known blood‐based marker that has demonstrated potential prognostic value in MIBC patients. It has been previously reported that high NLR was associated with a decreased response to NAC.^28^, ^29^ However, a secondary analysis of SWOG 8710 conducted by Ojerholm et al. showed no significant association between NLR and response to NAC.^30^ Thus, although tobacco history and NLR are simple risk factors and have been proved to be predictive of NAC response to some extent, well‐designed randomized phase III trials are needed to validate these results.

We then evaluated the dynamic alterations of TME after NAC, by comparing TILs and TAMs in paired MIBC tissues before and after NAC. Previous study showed decreased infiltration levels of CD8^+^ T cell and FOXP3^+^ Treg cell in the post‐NAC tissue.^23^ While in our study, the infiltration levels of CD4^+^ T cell, CD8^+^ T cell, or FOXP3^+^ Treg cell were determined not to change significantly after NAC. Notably, our results revealed that CD68^+^ and CD68^+^PD‐L1^−^ TAM infiltration levels decreased significantly after NAC, suggesting that the changes of CD68^+^ and CD68^+^PD‐L1^−^ TAMs, rather than CD4^+^ T cell or CD8^+^ T cell, might be the significant mechanism by which NAC regulates TME. It is well‐known that rejuvenating TILs is the major anti‐tumor mechanism of PD‐1/PD‐L1 blockade.^31^ Recent evidences show that there also exists complicated interaction between macrophage reprogramming and PD‐L1 expression.^32^ PD‐L1 is expressed on both M1‐like and M2‐like TAMs. Notably, it has been previous reported that macrophage reprogramming from M2‐like to M1‐like might increase the expression of PD‐L1.^32^, ^33^ NAC might play important roles in TAMs infiltrating level and macrophage reprogramming. Thus, the stable infiltration level CD68^+^PD‐L1^+^ TAMs in paired MIBC tissues before and after NAC implicates a potential rationale for the combination of PD‐L1 inhibitors with chemotherapy in the neoadjuvant setting.

Our study had some notable limitations. First, this was a single‐center retrospective research with relatively small patient cohort, further investigation based on multiple centers is expected. In addition, the multiplex immunofluorescence staining was restricted to CD4^+^, CD8^+^, CD68^+^, FOXP3^+^, and PD‐L1^+^. Several critical immune cell markers, including markers to identify M1‐like and M2‐like TAMs, were not examined.

## 5 CONCLUSION

Despite the limitations of this study, the results demonstrated that CD68^+^ and CD68^+^PD‐L1^−^ TAM infiltration levels decreased significantly after NAC and pre‐treatment TAM infiltration levels were independent prognostic factors for MIBC patients. This dynamic data suggest that NAC might affect TME through regulating the infiltration level CD68^+^ and CD68^+^PD‐L1^−^ TAMs. Unfortunately, there was no sufficient evidence demonstrating that pre‐treatment TILs or TAMs could predict response to NAC in MIBC patients.

## AUTHORS CONTRIBUTION

Jie Wu conceived the idea and was a major contributor in manuscript writing. Rui‐Yang Xie and Li‐Hui Wei conducted the data collection and was involved in the manuscript writing. Bing‐Qing Shang, Wang Qu, Yun Li, Jing Liang, and Shan Zheng performed all statistical analyses. You‐Yan Guan, Hong‐Zhe Shi, and Chuan‐Zhen Cao designed and performed the experiments. Xin‐Gang Bi, Jian‐Zhong Shou, Xiao‐Feng Zhou, and Ai‐Ping Zhou critically revised the manuscript. All authors read and approved the final version of manuscript.

## FUNDING INFORMATION

This work was supported by the CAMS Innovation Fund for Medical Sciences (NO. 2021‐I2M‐C&T‐B‐052).

## CONFLICT OF INTEREST

The authors declare no conflict of interest.

## ETHICS APPROVAL AND CONSENT TO PARTICIPATE

This study was approved by the institutional ethics committee of National Cancer Center/National Clinical Research Center for Cancer/Cancer Hospital, Chinese Academy of Medical Sciences and Peking Union Medical College, and written informed consent was obtained from all the patients preoperatively.

## CONSENT FOR PUBLICATION

Not applicable.

## Supporting information


Figure S1

Figure S2

Table S1

## Data Availability

All data generated or analyzed during this study are included in this published article.
