# Supplementary material for: Dynamic alteration and prognostic significance of tumor‐associated CD68+ and CD68+PD‐L1− macrophages in muscle‐invasive bladder cancer treated with neoadjuvant chemotherapy
Source: Cancer Med. 2022 Aug 31;12(4):4981–92. doi: 10.1002/cam4.5191 (PMC9972069; doi:10.1002/cam4.5191)
Supplement: Supplementary file 1 — Figure S1 [file CAM4-12-4981-s003.pdf]

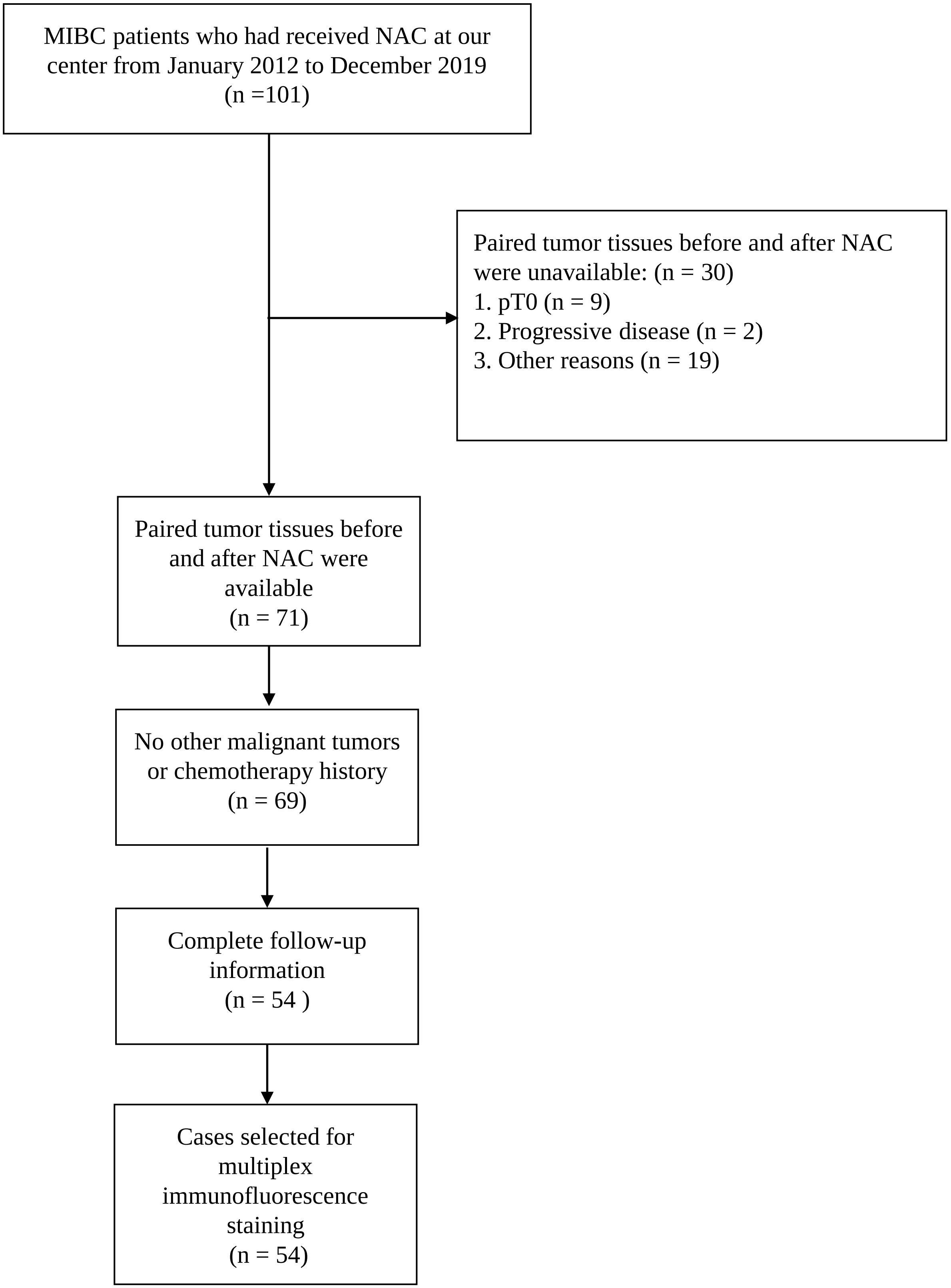
MIBC patients who had received NAC at our center from January 2012 to December 2019 (n =101)
Paired tumor tissues before and after NAC were unavailable: (n = 30)
1. pT0 (n = 9)
2. Progressive disease (n = 2)
3. Other reasons (n = 19)
Paired tumor tissues before and after NAC were available (n = 71)
No other malignant tumors or chemotherapy history (n = 69)
Complete follow-up information (n = 54 )
Cases selected for multiplex immunofluorescence staining (n = 54)
