# Supplementary figures and images for: Dynamic alteration and prognostic significance of tumor‐associated CD68+ and CD68+PD‐L1− macrophages in muscle‐invasive bladder cancer treated with neoadjuvant chemotherapy
Source: Cancer Med. 2022 Aug 31;12(4):4981–92. doi: 10.1002/cam4.5191 (PMC9972069; doi:10.1002/cam4.5191)

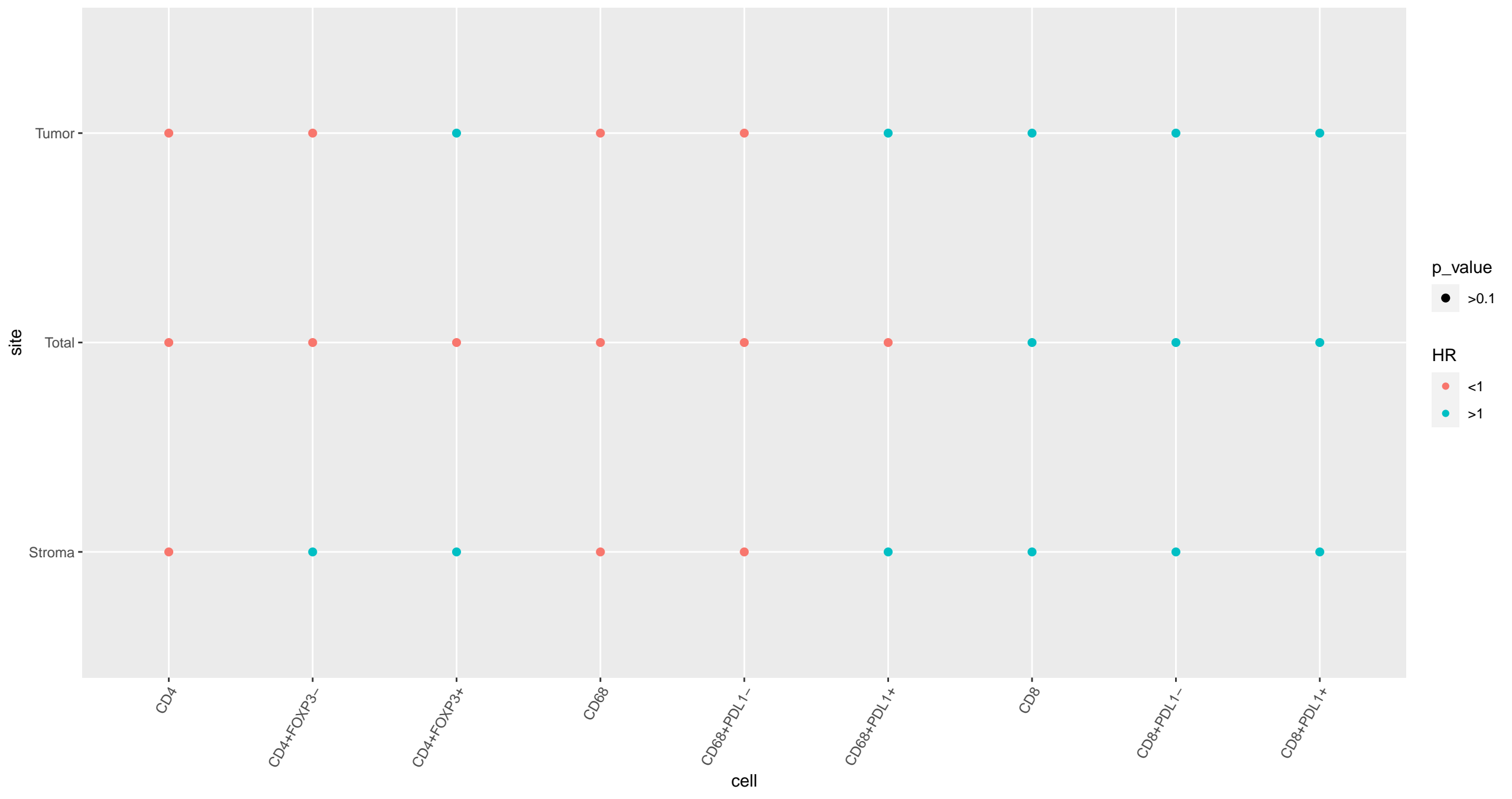

Supplement: Supplementary file 2 — Figure S2 [file CAM4-12-4981-s002.pdf]
