# Supplementary material for: Dynamic alteration and prognostic significance of tumor‐associated CD68+ and CD68+PD‐L1− macrophages in muscle‐invasive bladder cancer treated with neoadjuvant chemotherapy
Source: Cancer Med. 2022 Aug 31;12(4):4981–92. doi: 10.1002/cam4.5191 (PMC9972069; doi:10.1002/cam4.5191)
Supplement: Supplementary file 3 — Table S1 [file CAM4-12-4981-s001.docx]

**Supplementary Table** **1** Univariate logistic regression analysis of variables associated with NAC efficacy

|  | Univariate analysis | | |
| --- | --- | --- | --- |
|  | OR | 95%CI | *p* value |
| Age |  |  |  |
| ≤60 | Ref |  |  |
| >60 | 1.754 | 0.589-5.358 | 0.315 |
| Gender |  |  |  |
| Male | Ref |  |  |
| Female | 0.606 | 0.109-3.039 | 0.540 |
| **Tobacco History** |  |  |  |
| No | Ref |  |  |
| Yes | 3.405 | 1.098-11.345 | **0.038** |
| NLR |  |  |  |
| ≤3 | Ref |  |  |
| >3 | 2.824 | 0.861-10.395 | 0.097 |
| BMI |  |  |  |
| ≤25 | Ref |  |  |
| >25 | 0.834 | 0.277-2.497 | 0.745 |
| Pre-treatment T Stage |  |  |  |
| 2 | Ref |  |  |
| 3 | 1.188 | 0.339-4.180 | 0.786 |
| 4 | 1.500 | 0.189-14.248 | 0.701 |
| Lesion |  |  |  |
| Single lesion | Ref |  |  |
| Overlapping lesion | 0.488 | 0.156-1.467 | 0.205 |
| Tumor size, cm |  |  |  |
| ≤5 | Ref |  |  |
| >5 | 0.852 | 0.096-7.558 | 0.877 |

NAC: neoadjuvant chemotherapy; NLR: neutrophil-to-lymphocyte ratio; BMI: body mass index.

The bold values indicated that p < 0.05.
